# Similarities and discrepancies between commercially available bioelectrical impedance analysis system and dual-energy X-ray absorptiometry for body composition assessment in 10–14-year-old children

**DOI:** 10.1038/s41598-023-44217-0

**Published:** 2023-10-13

**Authors:** Kumiko Ohara, Harunobu Nakamura, Katsuyasu Kouda, Yuki Fujita, Tomoki Mase, Katsumasa Momoi, Toshimasa Nishiyama

**Affiliations:** 1 Department of Hygiene and Public Health, Kansai Medical University, 2-5-1 Shin-machi, Hirakata, Osaka 573-1010 Japan; 2 Graduate School of Human Development and Environment, Kobe University, 3-11 Tsurukabuto, Nada, Kobe, Hyogo 657-8501 Japan; 3 Center for Medical Education, Kindai University Faculty of Medicine, 377-2 Oono-Higashi, Osaka-Sayama, Osaka 589-8511 Japan; 4 Faculty of Human Development and Education, Kyoto Women’s University, 35 Imagumanokitahiyoshi-cho, Higashiyama, Kyoto, Kyoto 605-8501 Japan

**Keywords:** Epidemiology, Public health, Weight management

## Abstract

A variety of easy-to-use commercial bioelectrical impedance appliances are available. The aim of this study was to examine the usefulness of a commercially available body composition meter using bioelectrical impedance analysis (BIA) by comparing its measurement results with those obtained from dual-energy X-ray absorptiometry (DXA). The participants were 443 children aged from 10 to 14 years (226 boys and 217 girls). Fat mass, fat-free mass, lean body mass, percentage of body fat, and bone mineral contents were evaluated for all participants using BIA and DXA. The agreement in the anthropometric data obtained from both devices was analyzed using correlation analysis, intraclass correlation coefficient (ICC), Lin’s concordance correlation coefficient (CCC), Bland–Altman plots, and ordinary least products regression analysis. Equivalence between both devices was tested by two one-sided *t*-test. All measured indicators showed strong linear correlations between the two measurement systems (r, 0.853–1.000). Fat mass, fat-free mass, and lean body mass showed absolute concordance (ICC, 0.902–0.972; Lin’s CCC, 0.902–0.972). BIA overestimated bone mineral content (62.7–66.5%) and underestimated percentage of body fat (− 8.9 to − 0.8%), lean body mass (− 3.5 to − 1.8%), and body mass (− 0.8 to − 0.5%). For fat mass and fat-free mass, the overestimate or underestimate varied according to the sex and statistical analysis test. Bland–Altman analysis and ordinary least products analysis showed fixed bias and proportional bias in all indicators. Results according to quartiles of body mass index showed poor agreement for fat mass and percentage of body fat in both boys and girls in the lowest body mass index quartile. The present results revealed strong linear correlations between BIA and DXA, which confirmed the validity of the present single-frequency BIA-derived parameters. Our results suggest that BIA cannot provide the exact same values as DXA for some body composition parameters, but that performance is sufficient for longitudinal use within an individual for daily health management and monitoring.

## Introduction

Body mass index (BMI) is easily calculated from height and body weight and has been widely used in field studies as a simple indicator of obesity^1^. However, body weight represents the mass of the body as a whole, and as BMI is a ratio of mass to height, it does not necessarily provide information on the body composition; that is, the major components that make up the body, muscle, fat, and bone. Skeletal muscles play an important role in physical activity and postural maintenance^2,3^, and insufficient muscle mass may result in the risk of sarcopenia^4,5^. Insufficient bone mass increases the risk of osteoporosis, whereas high maximum bone mass acquired during growth prolongs the onset of osteoporosis^6^. Excessive body fat content is a risk for cardiovascular and other diseases^7–9^. In addition, a high percentage of body fat, even with a BMI within the normal range (which is considered normal weight obesity^10^), is reported to be associated with metabolic dysregulation^10^, metabolic syndrome^11^, and coronary heart diseases^12^. Thus, the normality of the body composition is critical to lifelong health. Therefore, obtaining information on body composition is very important for maintaining lifelong health, and if it can be easily obtained in daily life, it can be a useful tool for personal health management. To do that, body composition must be measured as accurately as possible.

Recently, dual-energy X-ray absorptiometry (DXA) and bioelectrical impedance analysis (BIA) have become used as methods for measuring body composition. DXA is used to evaluate body composition according to the amount of dual-energy X-rays absorbed, and is considered to be an accurate method for determining body composition^13,14^. In addition to the primary assessment of bone mineral content, the fat mass and lean mass can be calculated^15^. However, because of equipment costs, operating procedures, and logistical issues, the use of DXA in large-scale applied studies has not been encouraged^16^.

BIA measures resistance to current, and fat-free tissue has the lowest resistance because of its high-water content^17^. BIA has been proposed as a safe noninvasive simple portable quick and low-cost alternative to DXA, and it can provide acceptable body composition reports in terms of accuracy and reliability^16,18,19^. BIA devices have become more sophisticated in recent years and now include devices using multiple frequencies, which have been used in investigations on the similarity of body composition estimates between DXA and BIA^17,20–26^. However, although such multiple frequency bioelectrical impedance analyzers less expensive than DXA, but they are not necessarily intended for routine measurements in homes and schools.

In recent years, BIA devices have become commercially available for home use. These BIA devices make measurements using a single frequency and are inexpensive and easy to use, making them suitable for daily use. However, BIA measures the conductive properties of human tissues and is susceptible to the subject’s hydration state (extracellular water)^14^. Unlike multi-frequency BIA devices, the single-frequency BIA devices make measurements using only a low frequency of 50 kHz, which means that extracellular fluid can be measured, but not intracellular fluid, and thus the accuracy of the measurement is affected by fluctuations in water content distribution. As the number of available BIA devices increases, studies have demonstrated the effectiveness of both single-frequency BIA and multi-frequency BIA devices, and have suggested that BIA may be used as an alternative to DXA in body composition assessment^27,28^. However, accuracy differs widely across BIA devices, and single-frequency BIA seems to show larger differences than multi-frequency BIA^29,30^. Furthermore, even dual-frequency BIA can show large differences from DXA^31^. In studies of obese and diseased children, single-frequency BIA has shown discrepancies with DXA^32,33^. Impedance is a function of reactance and resistance^28,34^, and a potential solution to this problem is to incorporate technology that can measure reactance, making it possible for even a single-frequency BIA device to acquire information that reflects not only the resistance of the extracellular fluid, but also information from the cell membrane, thereby improving the accuracy of the measurements.

BIA provides body composition estimates based on a formula obtained using values measured by DXA as the reference. The reference populations used are primarily adults, and no estimates have been made using data from children. Therefore, how applicable the estimations made for adults are to children needs to be verified.

In this study, we examined the usefulness of a commercially available single-frequency BIA device incorporating reactance measurement technology for making body composition estimates in children, comparing its results with those obtained using DXA.

## Methods

### Overview

In this study, we tested similarities and discrepancies between a commercially available BIA device and a DXA scanner. The single-frequency BIA device provides body composition data in the form of body fat, body mass, and lean body mass estimates. BIA-derived body composition data were plotted against DXA scan data to determine criterion validity and measurement agreement. In addition, because it is reported that the accuracy of the BIA device is sex and age-dependent^35^, we considered subgroups of girls and boys separately.

### Participants

We conducted a population-based cross-sectional study in the Togan area of Awaji city in 2014, which is located in the suburban area of Hyogo prefecture in the central part of Japan, where four elementary schools and two junior high schools existed at the time. The participants were fifth and sixth graders who attended one of four public elementary schools, and first and second graders at two public junior high schools. The participants totaled 443 children, including 206 elementary school children (108 boys and 98 girls) and 237 junior high school children (118 boys and 119 girls). The study was approved by the Human Ethics Committee of the Graduate School of Human Development and Environment, Kobe University. All methods were performed in accordance with the relevant guidelines and regulations. Parents of all participants enrolled in this study provided informed consent.

### Procedures

The body composition of participants was measured by BIA after their body height and body mass were measured in a room in a school assigned exclusively for performing measurements, and immediately afterwards their body composition was measured by DXA. Body composition was measured at least one hour after any meal or exercise.

### Body height and body mass

Body mass was measured to the nearest 0.1 kg using an electronic height-weight scale (AD-6351, A & D Company Ltd., Tokyo, Japan), with participants wearing minimal clothing and without shoes. Body height was measured to the nearest 0.1 cm using an electronic height-weight scale (AD-6351), with the heels, buttocks, and upper back touching the posts while freestanding without shoes. BMI was calculated as body mass (kg) divided by body height (m) squared.

### Bioelectrical impedance analysis (BIA)

BIA was performed using a single-frequency (50 kHz) BIA system (BC-622, Tanita Corp., Tokyo, Japan). According to the manufacturer’s manual, measurements were taken with the participant’s bare feet on the electrode plate and their hands on both ends of the grips. When holding these grips, all fingers were placed over the electrodes on the grips. The participants lowered their arms straight down in front of their bodies so that their arms and the grips did not touch their bodies or legs. They were instructed not to bend their knees or elbows, and not to move during the measurement. Fat mass, fat-free mass, lean body mass, percentage of body fat, bone mineral contents, and body mass were determined according to the manufacturer’s standard operating procedures.

### Dual-energy X-ray absorptiometry (DXA)

DXA assessments were conducted using a single DXA scanner (QDR-4500A, Hologic Inc., Bedford, MA, USA) mounted in a mobile examination car that attended each school. Throughout the surveys, quality control of the DXA scanner was performed using the Step Phantom scan for body composition. The DXA considered variables were total body fat, lean body mass, and body mass. A single experienced radiological technologist performed all scans and scan analyses.

### Statistical analysis

Two-way analysis of variance (ANOVA) was used to investigate the effects of sex and grade and the interaction effects between sex and grade on the parameters of body height, body mass, and BMI. The Bonferroni test was used for post-hoc analysis. Paired *t* test was used to evaluate differences between BIA and DXA values. The two one-sided *t*-test (TOST) was used to test possible equivalence between BIA and DXA variables^36,37^. The assumed equivalence intervals were ± 1 kg in fat mass, fat-free mass, lean body mass, and body mass; ± 0.1 kg in body mineral contents; and ± 1% in percentage of body fat; in accord with previous reports^19,38^. The constant error (CE) was calculated by subtracting the value measured by DXA from the value measured by BIA. The standard error of estimate (SEE) was used to assess the statistical conformity of BIA as a predictor of DXA. The standard error of measurement (SEM) was first calculated then the minimum difference (MD) was calculated by multiplying the SEM by 1.96 multiplied by the square root of 2. MD represents the limits within which the observed difference is within what we might expect to see in repeated testing under only variation due to measurement noise^39^. The Pearson’s product-moment correlation was calculated between BIA and DXA measurements. The magnitude of the reported effects was described using the criteria of Hopkins et al.^40^ as follows: trivial, *r* < 0.1; small, 0.1 ≤ r < 0.3; moderate, 0.3 ≤ r < 0.5; large, 0.5 ≤ r < 0.7; very large, 0.7 ≤ r < 0.9; nearly perfect, *r* ≥ 0.9; and perfect, *r* = 1. The intraclass correlation coefficient (ICC; two-way random and single measure) was used to assess agreement between BIA and DXA^41^. Intraclass correlations of < 0.5, 0.5 ≤ and ≤ 0.75, 0.75 < and 0.90, and > 0.90 were considered indicative of poor, moderate, good, and excellent repeatability, respectively^42^. Lin’s concordance correlation coefficient (CCC) was used to assess how close the BIA and DXA data were around the line of best fit, and also how far that line was from the 45-degree line through the origin^43^. Interpretation of Lin’s CCC (ρ_c_) was followed by McBride proposal: ρ_c_ ≥ 0.99, almost perfect; 0.99 > ρ_c_ ≥ 0.95, substantial; 0.95 > ρ_c_ ≥ 0.9, fair; ρ_c_ < 0.9, poor^44^. Bland–Altman analysis was used to identify the 95% limits of agreement (LOA), which were calculated to assess the agreement between BIA and DXA^45^. Following the Bland–Altman analysis, a one-sample *t* test with a test value of 0 was used to evaluate fixed effects. In addition, regression analysis was used to evaluate proportional errors. Ordinary least products (OLP) regression analysis was used to determine the proportional bias and fixed bias^46^. Fixed bias was considered to be present if the 95% confidence interval (CI) of the intercept (x) did not cross 0, and proportional bias was also considered to be present if the 95% CI of the slope (y) did not cross 1.0^47,48^. The level of statistical significance was set at 0.05. All statistical analyses were performed with SPSS 28.0 for Windows (International Business Machines Corp, Armonk, NY, USA) and Jamovi 2.3.18.0 for Windows (The Jamovi Project, Sydney, Australia).

## Results

The height, body mass, and BMI of the participants in each grade are shown in Table 1. Height and body mass showed significant interactions between sex and grade. Grade showed main effects on height, body mass, and BMI.

**Table 1:** Participant height, body weight, and body mass index.

	Age (years)	Height*^,†^ (cm)	Body mass*^,†^ (kg)	Body mass index* (kg/m^2^)
Boys				
5th grade (n = 55)	10.7 ± 0.5 (10–11)	141.2 ± 5.9 (127.0–157.2)	35.3 ± 8.2 (25.1–63.9)	17.6 ± 3.1 (14.0–30.3)
6th grade (n = 53)	11.5 ± 0.5 (11–12)	146.3 ± 8.3^§,‡^ (130.4–165.2)	38.3 ± 9.7 (25.1–71.2)	17.7 ± 3.0^‡^ (14.0–30.9)
7th grade (n = 63)	12.6 ± 0.5 (12–13)	154.9 ± 9.3^‡,‖^ (133.9–173.0)	47.5 ± 13.4^§,‡,‖^ (28.2–97.5)	19.5 ± 3.9^‖^ (14.2—32.6)
8th grade (n = 55)	13.6 ± 0.5 (13–14)	161.5 ± 7.3^§,‡,‖,⁋^ (146.0–176.4)	49.4 ± 9.0^‡,‖^ (28.8–66.9)	18.8 ± 2.4 (12.1–26.0)
All boys (n = 227)	12.6 ± 0.5 (10–13)	151.2 ± 11.0 (127.0–176.4)	42.8 ± 11.9 (25.1–97.5)	18.4 ± 3.3 (12.1–32.6)
Girls				
5th grade (n = 48)	10.6 ± 0.5 (10–11)	142.8 ± 7.3 (128.2–156.6)	35.5 ± 7.2 (23.4–50.9)	17.2 ± 2.3 (13.7–23.0)
6th grade (n = 50)	11.5 ± 0.5 (11–12)	149.1 ± 6.4^‡^ (134.4 – 164.7)	40.7 ± 8.3^‡^ (22.2–60.8)	18.2 ± 2.8 (11.5–26.2)
7th grade (n = 73)	12.6 ± 0.5 (12–13)	152.8 ± 5.2^‡,‖^ (139.4–163.6)	43.6 ± 6.6^‡^ (31.7–62.7)	18.6 ± 2.2 (15.0–25.5)
8th grade (n = 46)	13.6 ± 0.5 (13–14)	154.5 ± 5.1^‡,‖^ (139.1–163.7)	46.1 ± 7.2^‡,‖^ (34.1–68.2)	19.3 ± 2.5^‡^ (15.2–26.3)
All girls (n = 217)	13.6 ± 0.5 (12–13)	150.1 ± 7.3 (128.2–164.7)	41.7 ± 8.2 (22.2–68.2)	18.4 ± 2.5 (11.5–26.3)

Data are mean ± standard deviation. Ranges are in the parenthesis.

*Significant main effect of grade (p < 0.05, Two-way analysis of variance).

^†^Significant interaction effect between sex and grade (p < 0.05, Two-way analysis of variance).

^§^Significantly different from girls in the corresponding grade (p < 0.05, Bonferroni test for post-hoc test).

^‡^Significantly different from 5th grade (p < 0.05, Bonferroni test for post-hoc test).

^‖^Significantly different from 6th grade (p < 0.05, Bonferroni test for post-hoc test).

^⁋^Significantly different from 7th grade (p < 0.05, Bonferroni test for post-hoc test).

The measurement values of BIA and DXA, and their CE, SEE, and MD are shown in Table 2. Lean mass, bone mineral contents, and body mass measurements showed a significant difference between BIA and DXA according to paired t test. According to the constant error, bone mineral contents were overestimated on BIA in boys, girls, and all participants; the percentage of body fat, lean body mass, and body mass were underestimated in boys, girls, and all participants; fat mass was underestimated in boys and all participants but overestimated in girls; and fat-free mass was overestimated in boys but underestimated in girls and all participants.

**Table 2:** Fat mass, fat-free mass, percentage of body fat, lean body mass, bone mineral contents, and body mass by BIA and DXA.

	BIA	DXA	CE (95% CI)	SEE	MD
Boys (n = 226)					
FM (kg)	7.6 ± 6.2	7.9 ± 4.4	− 0.28 ± 2.37 (− 0.59, 0.03)	1.284	1.36
FFM (kg)	35.7 ± 7.6	35.7 ± 8.8	0.07 ± 2.01 (− 0.19, 0.34)	1.704	0.77
PBF (%)	16.0 ± 8.3	17.6 ± 6.1*	− 1.57 ± 4.47 (− 2.16, − 0.99)	3.207	4.75
LBM (kg)	33.9 ± 7.1	34.5 ± 8.5*	− 0.63 ± 2.08 (− 0.90, − 0.35)	1.681	0.82
BMC (kg)	1.8 ± 0.5	1.1 ± 0.3*	0.70 ± 0.20 (0.68, 0.73)	0.115	0.15
BM (kg)	43.3 ± 11.9	43.6 ± 11.5*	− 0.21 ± 0.48 (− 0.27, − 0.14)	0.310	0.00
Girls (n = 217)					
FM (kg)	9.4 ± 4.2	9.4 ± 3.6	0.01 ± 1.24 (− 0.16, 0.17)	0.963	0.66
FFM (kg)	32.8 ± 4.5	33.1 ± 5.2*	− 0.36 ± 1.09 (− 0.51, − 0.21)	0.918	0.38
PBF (%)	21.4 ± 5.9	21.5 ± 4.9	− 0.18 ± 2.81 (− 0.55, 0.20)	2.321	2.70
LBM (kg)	30.9 ± 4.2	32.0 ± 5.0*	− 1.11 ± 1.15 (− 1.27, − 0.96)	0.904	0.41
BMC (kg)	1.9 ± 0.3	1.1 ± 0.3*	0.76 ± 0.17 (0.74, 0.78)	0.119	0.16
BM (kg)	42.2 ± 8.1	42.5 ± 7.9*	− 0.35 ± 0.32 (− 0.39, − 0.31)	0.263	0.03
All (n = 443)					
FM (kg)	8.5 ± 5.4	8.6 ± 4.1	− 0.14 ± 1.91 (− 0.32, 0.04)	1.187	1.10
FFM (kg)	34.3 ± 6.4	34.4 ± 7.4	− 0.14 ± 1.64 (− 0.29, 0.14)	1.433	0.63
PBF (%)	18.6 ± 7.7	19.5 ± 5.9*	− 0.89 ± 3.81 (− 1.25, − 0.53)	2.830	3.70
LBM (kg)	32.4 ± 6.0	33.3 ± 7.1*	− 0.87 ± 1.7 (− 1.02, − 0.71)	1.440	0.69
BMC (kg)	1.9 ± 0.4	1.1 ± 0.3*	0.73 ± 0.19 (0.71, 0.75)	0.117	0.16
BM (kg)	42.8 ± 10.2	43.1 ± 9.9*	− 0.28 ± 0.42 (− 0.32, − 0.24)	0.295	0.00

Data are means ± standard deviations.

BIA, bioelectrical impedance analysis; DXA, dual energy X-ray absorptiometry; CE, constant error (BIA minus DXA value); CI, confidence interval; SEE, standard error of estimate; MD, minimum difference; FM, fat mass; FFM, fat-free mass; PBF, percentage of body fat; LBM, lean body mass; BMC, bone mineral contents; BM, body mass.

*p < 0.05 (Paired t test between BIA and DXA).

The Pearson’s correlation coefficients, ICCs, and Lin’s CCCs are shown in Table 3. The Pearson’s correlation coefficients between BIA and DXA were perfect for body mass of boys and all participants; nearly perfect for fat mass, fat-free mass, and lean body mass of boys, girls, and all participants and body mass of girls and bone mineral contents of boys and all participants; and very large for percentage of body fat of boys, girls, and all participants and bone mineral contents of girls. The ICCs were excellent for fat mass, fat-free mass, lean body mass, and body mass of boys, girls, and all participants; good for percentage of body fat of boys, girls, and all participants; and poor for bone mineral contents of boys, girls, and all participants. The Lin’s CCCs were almost perfect for body mass of boys, girls, and all participants; substantial for fat-free mass of boys, girls, and all participants and lean body mass of boys and all participants; fair for fat mass of boys, girls, and all participants and lean body mass of girls; and poor for percentage of body fat and bone mineral contents of boys, girls, and all participants.

**Table 3:** Pearson’s correlation coefficients, ICC, and Lin’s CCC of body composition between BIA and DXA.

	r (95% CI)	ICC (2.1) (95% CI)	Lin’s CCC (95%CI)
Boys (n = 226)			
FM (kg)	0.957 (0.944 to 0.967)	0.902 (0.874 to 0.924)	0.902 (0.885 to 0.916)
FFM (kg)	0.981 (0.976 to 0.985)	0.970 (0.961 to 0.977)	0.970 (0.963 to 0.976)
PBF (%)	0.853 (0.812 to 0.885)	0.796 (0.713 to 0.852)	0.823 (0.781 to 0.859)
LBM (kg)	0.980 (0.975 to 0.985)	0.962 (0.946 to 0.972)	0.962 (0.953 to 0.968)
BMC (kg)	0.929 (0.909 to 0.945)	0.329 (− 0.037 to 0.697)	0.328 (0.283 to 0.371)
BM (kg)	1.000 (1.000 to 1.000)	0.999 (0.998 to 0.999)	0.999 (0.999 to 0.999)
Girls (n = 217)			
FM (kg)	0.963 (0.952 to 0.971)	0.949 (0.934 to 0.961)	0.949 (0.937 to 0.959)
FFM (kg)	0.984 (0.980 to 0.988)	0.972 (0.959 to 0.981)	0.972 (0.966 to 0.978)
PBF (%)	0.880 (0.846 to 0.907)	0.865 (0.827 to 0.895)	0.876 (0.841 to 0.903)
LBM (kg)	0.983 (0.978 to 0.987)	0.941 (0.662 to 0.978)	0.941 (0.928 to 0.952)
BMC (kg)	0.883 (0.850 to 0.910)	0.203 (− 0.022 to 0.552)	0.202 (0.168 to 0.235)
BM (kg)	0.999 (0.999 to 1.000)	0.998 (0.998 to 0.999)	0.998 (0.998 to 0.999)
All (n = 443)			
FM (kg)	0.957 (0.948 to 0.964)	0.920 (0.905 to 0.933)	0.920 (0.909 to 0.930)
FFM (kg)	0.981 (0.977 to 0.984)	0.972 (0.966 to 0.977)	0.972 (0.964 to 0.976)
PBF (%)	0.877 (0.853 to 0.897)	0.839 (0.802 to 0.869)	0.859 (0.835 to 0.881)
LBM (kg)	0.979 (0.975 to 0.983)	0.958 (0.920 to 0.975)	0.958 (0.952 to 0.964)
BMC (kg)	0.910 (0.893 to 0.925)	0.269 (− 0.030 to 0.635)	0.269 (0.240 to 0.296)
BM (kg)	1.000 (0.999 to 1.000)	0.999 (0.996 to 0.999)	0.999 (0.999 to 0.999)

ICC, intraclass correlation coefficients; CCC, concordance correlation coefficients; BIA, bioelectrical impedance analysis; DXA, dual energy X-ray absorptiometry; r, Pearson’s correlation coefficients; CI, confidence interval; FM, fat mass; FFM, fat-free mass; PBF, percentage of body fat; LBM, lean body mass; BMC, bone mineral contents; BM, body mass.

**Figure 1:**
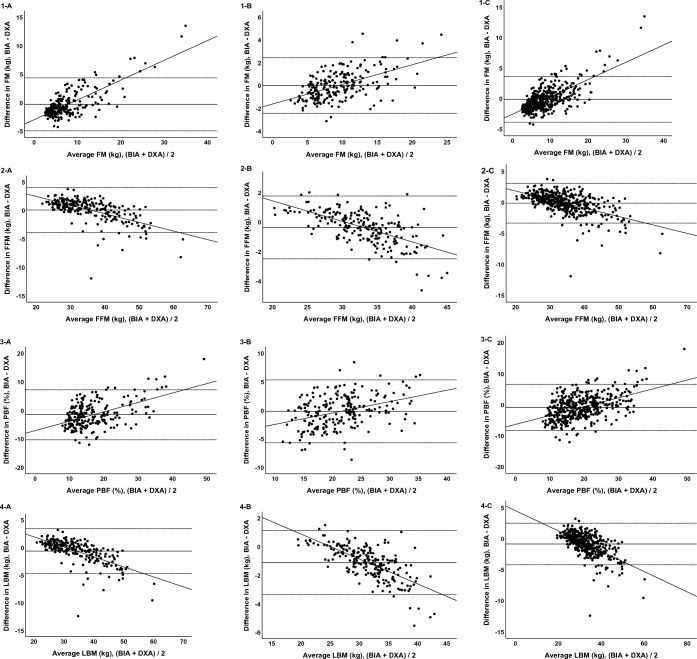

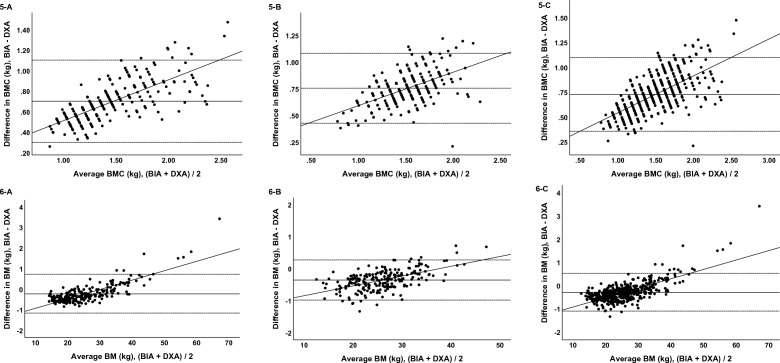
Bland–Altman plot of body composition data obtained with bioelectrical impedance-based methods (BIA) and dual-energy Xray absorption method (DXA). (**1-A**) fat mass in boys, (**1-B**) fat-mass in girls, (**1-C**) fat-mass in all participants, (**2-A**) fat-free mass in boys, (**2-B**) fat-free mass in girls, (**2-C**) fat-free mass in all participants,  (**3-A**) Percentage of body fat in boys, (**3-B**) percentage of body fat in girls, (**3-C**) percentage of body fat in all participants, (**4-A**) lean body mass in boys, (**4-B**) lean body mass in girls, (**4-C**) lean body mass in all participants, (**5-A**) Bone mineral contents in boys, (**5-B**) bone mineral contents in girls, (**5-C**) bone mineral contents in all participants, (**6-A**) body mass in boys, (**6-B**) body mass in girls, (**6-C**) body mass in all participants. BIA, bioelectrical impedance analysis; DXA, dual energy X-ray absorptiometry; FM, fat mass; FFM, fat-free mass; PBF, percentage of body fat; LBM, lean body mass; BMC, bone mineral contents; BM, body mass.

The results of the Bland–Altman analysis are shown in Table S1 and Fig. 1. The 95% LOA were ± 4.64 kg (boys) and ± 2.44 kg (girls) for fat mass, ± 3.93 kg (boys) and ± 2.14 kg (girls) for fat-free mass, ± 8.75% (boys) and ± 5.51% (girls) for percentage of body fat, ± 4.08 kg (boys) and ± 2.25 kg (girls) for lean body mass, ± 0.40 kg (boys) and ± 0.33 kg (girls) for bone mineral contents, and ± 0.94 kg (boys) and ± 0.63 kg (girls) for body mass. Fixed bias was found in lean body mass, bone mineral contents, and body mass in boys, girls, and all participants, fat-free mass in girls, and percentage of body fat in boys and all participants. Proportional bias was found to affect all parameters.

A summary of the OLP regression analysis for DXA and BIA is provided in Table 4. The OLP regression analysis revealed a fixed bias in all cases except for percentage of body fat and lean body mass of boys. Proportional bias was also detected in all cases except for percentage of body fat and lean body mass of boys.

**Table 4:** Ordinary least products regression analysis between BIA and DXA.

	a	95% CI for a	b	95% CI for b	Fixed bias*	Proportional bias*
Boys (n = 226)						
FM (kg)	− 3.518	− 4.003, − 3.033	1.410	1.356, 1.463	Yes	Yes
FFM (kg)	5.068	4.264, 5.871	0.860	0.838, 0.882	Yes	Yes
PBF (%)	− 0.855	− 2.144, 0.434	0.979	0.912, 1.046	No	No
LBM (kg)	− 0.626	− 1.546, 0.293	1.000	0.974, 1.026	No	No
BMC (kg)	0.168	0.085, 0.252	1.477	1.406, 1.549	Yes	Yes
BM (kg)	− 1.585	− 1.585, − 1.585	1.032	1.032, 1.032	Yes	Yes
Girls (n = 217)						
FM (kg)	− 1.729	− 2.158, − 1.300	1.185	1.142, 1.228	Yes	Yes
FFM (kg)	3.935	3.239, 4.631	0.870	0.850, 0.891	Yes	Yes
PBF (%)	− 2.380	− 3.934, − 0.825	1.099	1.029, 1.169	Yes	Yes
LBM (kg)	3.992	3.323, 4.661	0.840	0.820, 0.861	Yes	Yes
BMC (kg)	0.364	0.266, 0.463	1.345	1.261, 1.430	Yes	Yes
BM (kg)	− 1.307	− 1.572, − 1.043	1.022	1.142, 1.029	Yes	Yes
All (n = 443)						
FM (kg)	− 2.930	− 3.272, − 2.588	1.323	1.287, 1.359	Yes	Yes
FFM (kg)	4.227	3.670, 4.784	0.873	0.857, 0.889	Yes	Yes
PBF (%)	− 4.457	− 5.550, − 3.365	1.192	1.138, 1.245	Yes	Yes
LBM (kg)	4.126	3.575, 4.676	0.850	0.834, 0.866	Yes	Yes
BMC (kg)	0.243	0.178, 0.308	1.432	1.376, 1.487	Yes	Yes
BM (kg)	− 1.530	− 1.650, − 1.410	1.029	1.026, 1.032	Yes	Yes

BIA, bioelectrical impedance analysis; DXA, dual energy X-ray absorptiometry; CI, confidence interval; FM, fat mass; FFM, fat-free mass; PBF, percentage of body fat; LBM, lean body mass; BMC, bone mineral contents; BM, body mass.

*a and b: coefficients in ordinary least products regression model: E(A) = a + b(B) (a, (y axis) intercept; b, slope).

*Fixed bias, if 95% CI for a does not include 0; proportional bias, if 95% CI for b does not include 1.

**Figure 2:**
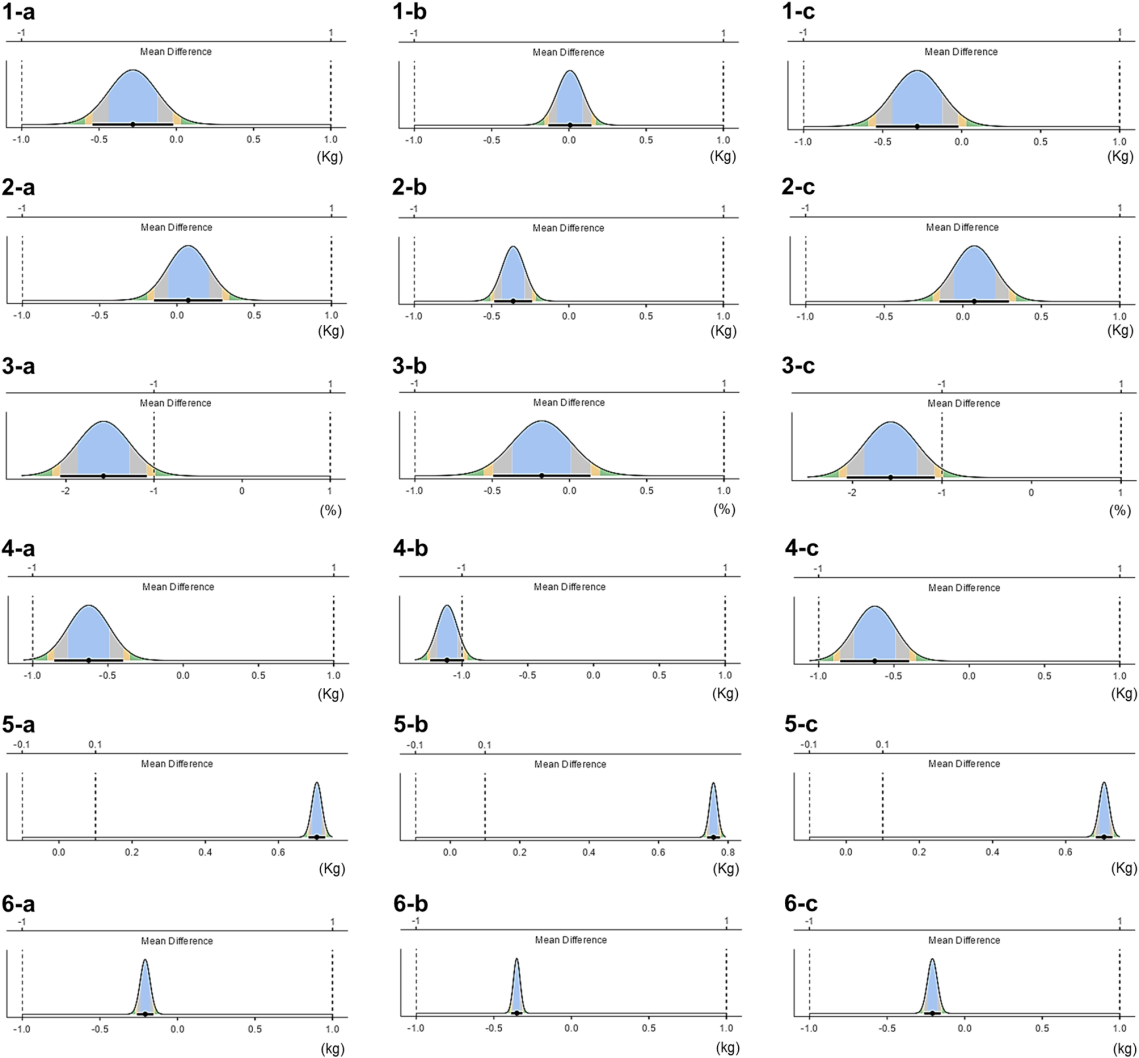
Two one-sided test of fat mass, fat-free mass, percentage of body fat, lean body mass, bone mineral contents, and body mass in boys. Black circles (●) indicate mean differences and black horizontal bars indicate 90% confidence intervals. (**1-a**) Fat mass in boys, (**1-b**) fat mass in girls, (**1-c**) fat mass in all participants, (**2-a**) fat-free mass in boys, (**2-b**) fat-free mass in girls, (**2-c**) fat-free mass in all participants, (**3-a**) percentage of body fat in boys, (**3-b**) percentage of body fat in girls, (**3-c**) percentage of body fat in all participants, (**4-a**) Lean body mass in boys, (**4-b**) lean body mass in girls, (**4-c**) lean body mass in all participants, (**5-a**) bone mineral contents in boys, (**5-b**) bone mineral contents in girls, (**5-c**) bone mineral contents in all participants, (**6-a**) body mass in boys, (**6-b**) body mass in girls, (**6-c**) body mass in all participants.

Difference plots across the variables from the TOST are reported in Fig. 2. In both boys and girls, the values of fat mass, fat-free mass, percentage of body fat, and lean body mass were between lower and upper bounds.

A summary of the statistical analysis is presented in Table 5. BIA overestimated bone mineral contents (boys, 62.7%; girls, 66.5%; all, 64.6%) and underestimated percentage of body fat (boys, − 8.9%; girls, − 0.8%; all, − 4.6%), lean body mass (boys, − 1.8%; girls, − 3.5%; all, − 2.6%), and body mass (boys, − 0.5%; girls, − 0.8%; all, − 0.6%). For fat mass and fat-free mass, the overestimate or underestimate varied depending on the sex and statistical analysis method.

**Table 5:**
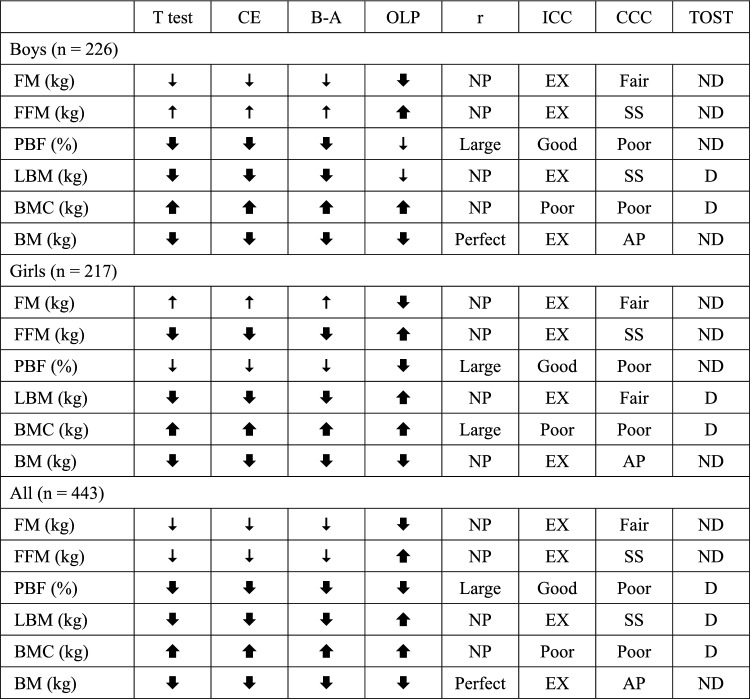
Summary of the statistics analysis in the present study.

BIA, bioelectrical impedance analysis; DXA, dual energy X-ray absorptiometry; T test, Paired t test; CE, constant error (BIA minus DXA value); B-A, Bland Altman analysis; OLP, ordinary least products regression analysis; r, Pearson’s product moment correlation coefficient; ICC, intraclass correlation coefficients; CCC, Lin’s concordance correlation coefficients; TOST, two one-sided t-test; FM, fat mass; FFM, fat-free mass; PBF, percentage of body fat; LBM, lean body mass; BMC, bone mineral contents; BM, body mass; NP, nearly perfect; EX, excellent; SS, substantial; AP, almost perfect; ND, not different; D, different.

significant overestimate,  significant underestimate,  overestimate but not significant,  underestimate but not significant.

Fat mass, fat-free mass, percentage of body fat, lean body mass, bone mineral contents, and body mass are shown according to BMI category for boys in Table S2 and for girls in Table S3. In the 2nd, 3rd, and 4th quartiles, Pearson’s correlation coefficients between BIA and DXA were nearly perfect or very large for all parameters in both boys and girls. In the 1st quartile, Pearson’s correlation coefficients between BIA and DXA were nearly perfect or very large for all parameters in both boys and girls, except for percentage of body fat in boys and girls and fat mass in boys. The ICCs were excellent or good for fat-free mass, lean body mass, and body mass in all quartiles in both boys and girls. The ICCs for bone mineral content were poor in all quartiles in both boys and girls. The Lin’s CCCs were substantial or fair for fat-free mass and body mass in all quartiles in both boys and girls, and for lean body mass in 1st, 2nd, and 3rd quartiles in boys, and 1st and 2nd quartiles in girls. The Lin’s CCCs were poor for fat mass, percentage of body fat, and bone mineral content in all quartiles in both boys and girls. Bland–Altman plots of each BMI category are shown in Figs. S1–S4 for both sexes.

Fat mass, fat-free mass, percentage of body fat, lean body mass, bone mineral contents, and body mass are shown according to grade for boys in Table S4 and for girls in Table S5. In all grades, Pearson’s correlation coefficients between BIA and DXA were nearly perfect or very large for all parameters in both boys and girls. The ICCs were excellent or good for fat mass, fat-free mass, percentage of body fat, lean body mass, and body mass in both boys and girls, except for percentage of body fat in 8th grade boys. The ICCs for bone mineral content were poor in all grades in both boys and girls. The Lin’s CCCs were substantial or fair for fat mass, fat-free mass, and body mass in all grades in both boys and girls except for fat mass in 6th and 8th grade boys and fat-free mass in 5th grade boys. The Lin’s CCC were also substantial or fair for lean body mass in 6th, 7th, and 8th grade boys and 5th, 6th, and 7th grade girls. The Lin’s CCCs were poor for percentage of body fat, and bone mineral content in all grades in both boys and girls. Bland–Altman plots of each grade are shown in Figs. S5–S8 for both sexes.

## Discussion

The purpose of this study was to evaluate the similarities and discrepancies of children’s body composition estimates between a commercially available single-frequency BIA device and DXA. Our results showed significant differences in the values of all parameters between BIA and DXA, with this being the case for boys, girls, and all participants. In addition, BIA overestimated bone mineral contents for boys, girls, and all participants, and underestimated lean body mass, body mass, and percentage of body fat. However, the differences in fat mass, fat-free mass, lean body mass, bone mineral contents, and body mass were within 1 kg for boys, girls, and all participants, with the exception of lean body mass for girls, which showed a small difference of 1.11 kg. Individual errors were also small, with SEE being less than 2 kg for fat mass, fat-free mass, and lean body mass, less than 4% for percentage of body fat, and less than 0.4 kg for bone mineral contents and body mass. Lee et al. performed DXA and single-frequency and multi-frequency BIA measurements on children aged 7–12 years and reported significant differences in lean body mass, fat mass, and percentage of body fat between single frequency BIA, multi-frequency BIA, and DXA^49^. BIA overestimated lean body mass, and underestimated fat mass and percentage of body fat^49^. Larsen et al. measured fat mass and lean body mass with multi-frequency BIA and DXA in children aged 10–12 years and reported that the measurements were significantly different between BIA and DXA, and that BIA underestimated both fat mass and lean body mass^38^. The results of the present study also revealed significant differences between DXA and BIA, consistent with previous results. Regarding the magnitude of the differences in measurements between DXA and single-frequency BIA, Lee et al. reported differences of lean body mass, fat mass, and percentage of body fat of 2.1 kg, 3.0 kg, and 4.8%, respectively, in boys, and 2.4 kg, 3.3 kg, and 9.7%, respectively, in girls^49^. Compared with these results, the differences between DXA and BIA measurements found in our study are small.

Larsen et al. reported differences between DXA and multi-frequency BIA measurements of 2.56 kg for boys and 2.33 kg for girls for fat mass, and 0.35 kg for boys and − 0.01 kg for girls for body mass^38^. Lee et al. reported differences in the mean lean body mass, fat mass, and percentage of body fat between DXA and BIA measurements of 0.2 kg, 1.3 kg, and 3.0%, respectively, in boys, and 0.4 kg, 1.7 kg, and 4.5% in girls^49^. The differences between multi-frequency BIA and DXA found in our study are comparable to these previous results.

The Pearson’s product moment correlation coefficients between DXA and BIA were above 0.9 for many indices, indicating strong linear correlations. When we checked the concordance of the ICCs and CCCs, we found that the values for fat mass, fat-free mass, lean body mass, and body mass were all more than 0.9. Lee et al. reported that for boys and girls, the ICCs between single or multi-frequency BIA and DXA for lean body mass, fat mass, and percentage of body fat were all above 0.9, except for a lean body mass value of 0.887 for single frequency BIA in boys^49^. In addition, all CCCs were above 0.9 except for the single frequency BIA percentage of body fat for boys, and the single and multi-frequency BIA percentage of body fat for girls^49^. In previous studies, CCCs for fat mass and fat-free mass in children were above 0.9, similar values to those found in the present results^50–52^. For percentage of body fat, the CCCs ranged from 0.747 to 0.881 between single or dual-frequency BIA and DXA, and from 0.912 to 0.926 between multi-frequency BIA and DXA^52^. A study by Seo et al. with Korean boys and girls (6–17 years) showed similar results^51^. Compared with the results of these previous studies, the BIA measurements obtained in this study showed a strong linear relationship with DXA and a high degree of agreement for fat mass, fat-free mass, and lean body mass.

In this study, we also used the equivalence testing method^36,53^ to assess measurement agreement. Previously, only Larsen et al. performed TOSTs to evaluate the agreement of fat mass between DXA and multi-frequency BIA in children^38^. In their study, they used the same equivalence interval as we did^19,38^, but while we found that the values of fat mass, fat-free mass, and body mass in both sexes, lean body mass in boys and percentage of body fat in girls, were between lower and upper bounds, the multi-frequency BIA used in the previous study found no agreement^38^. These results indicate that the agreement between DXA and the single-frequency BIA used in the present study was not inferior to that between DXA and multi-frequency BIA. Furthermore, in the Bland–Altman analysis, the limits of agreement were similar or smaller than in previous studies^19,20,54^. In contrast, most of the indicators showed fixed and proportional bias, and many indices show fixed biases and proportional biases in the results of the OLP regression analysis. The fact that both biases were observed in Bland–Altman analysis and OLP regression analysis indicates that absolute agreement was not always obtained, and that the values measured by BIA are not exact substitutes for DXA values. However, the small limits of agreement in the Bland–Altman analysis suggest that BIA can be used for health management purposes in daily life if the characteristics observed in this study are taken into account.

In contrast to the parameters mentioned above, bone mineral contents showed low ICCs, CCCs, and absolute agreements. For percentage of body fat, the ICCs or CCCs were below 0.9, showing lower absolute agreement than the other indices. However, the absolute agreements for fat mass, fat-free mass, lean body mass, and body mass were good, indicating that these BIA indices are reliable enough for daily use.

In addition, BIA overestimated bone mineral content and underestimated percentage of body fat, lean body mass, and body mass. For fat mass and fat-free mass, the overestimate or underestimate varied depending on the sex and statistical analysis method. Chiplonkar et al. reported that BIA overestimated fat mass, fat-free mass, and lean mass compared with DXA whereas underestimated percentage body fat^55^.Lopez-Gonzalez et al. reported that BIA overestimated fat-free mass, but underestimated fat mass and percentage of body fat relative to DXA^50^. Seo et al. reported that BIA overestimated fat-free mass and underestimated percentage of body fat and fat mass compared with DXA^51^. From these studies, overestimation or underestimation may depends on the particular BIA device used to make the measurements.

In BIA, fat-free mass is estimated from electrical resistance values, from which fat mass, percentage of body fat, bone mineral contents, and lean body mass are calculated^28^. Therefore, while accurate fat-free mass values are obtained directly, the other values are estimated from these fat-free mass values. The BIA device utilized in this study used a single-frequency of 50 kHz, which may result in inaccurate estimates of other indices compared with fat-free mass, because the evaluation of extracellular fluid and intracellular fluid is inferior compared with multi-frequency BIA^28^. However, we also showed that our measurement results were not inferior to those of other studies using multi-frequency BIA. This may be because the BIA device that we used utilizes an eight-pole electrode, and although it is a single frequency device, it uses reactance technology that allows impedance to be broken down into resistance and reactance, making it more accurate than conventional four-pole electrode BIA.

The DXA method uses the rate of attenuation of radiation irradiated to a living body to quantify bone mineral content^56^, whereas BIA measures electrical resistance that does not directly quantify bone mineral content^28^. In addition, recent BIA measurement methods are based on a multi-compartment model that calculates extracellular fixations, excluding body fat, body cell mass, and extracellular fluid^14,57^. In BIA, extracellular fixations are indicated as bone mineral content, which is the reason why BIA overestimates bone mineral contents in comparison with DXA. Therefore, compared with other indices, the bone mineral contents showed a greater discrepancy between BIA and DXA measurements, and also showed lower absolute concordance values such as ICC and CCC. However, relative concordance was found, and a considerable degree of relative concordance was obtained in sub group analyses by sex and BMI, and because BIA overestimated values, it would be possible not to provide clinical information but to observe bone mineral contents to a certain extent using the same BIA instrument for the purpose of daily health management.

The participants in this study had a wide range of BMI, and we therefore divided them into four categories according to sex and BMI and performed separate analyses in each category. The results showed a high relative agreement for all indicators in the top three BMI categories of both sexes. However, the relative agreement for percentage of body fat was low in the lowest BMI category for both boys and girls. In terms of absolute concordance, both ICCs and CCCs showed high concordance for body mass, fat-free mass, and lean body mass in most categories, although absolute agreement was low for bone mineral contents, in contrast to the relatively good relative agreement observed. Bland–Altman analysis of each index according to sex and BMI showed fixed and proportional bias for many indices, indicating that while the impedance method generally provides relative agreement, absolute agreement is limited to body mass and fat-free mass. The results for fat mass and percentage of body fat showed particularly poor agreement in the low BMI category. Because body mass measures total mass regardless of body composition, it is easy to make accurate measurements with commercially available scales, which explains the high concordance in body mass shown in this study.

We also performed separate analyses in each grade. The results showed the high relative agreement for all indicators in all grades of both sexes. In terms of absolute concordance, both ICCs and CCCs showed high concordance for fat mass, fat-free mass, lean body mass, and body mass in most grades, although absolute agreement was low for bone mineral contents. Bland–Altman analysis of each index according to sex and grade showed fixed and proportional bias for many indices. These results are similar to those obtained in the analysis by BMI category. However, there were no distinct differences in results between grades whereas there were differences in results between BMI categories. Total body water (TBW) is one of most important factors for BIA analysis. TBW changes according to the height, body mass, body surface area, and age. However, the association of TBW with height, body mass, and body surface are strong compared to that with age^58^, which may explain the present results.

In the present study, as in many other studies, DXA was used as the reference with which the BIA method was compared. However, the algorithm used in DXA is based on adult proportions and may not be accurate in children^59^. This is because the water content and body density of the fat-free mass of children is different to that of adults and changes throughout growth^59^. Therefore, to use DXA as a reference value for measurements in children, the DXA algorithm should be based on the proportions of the child, and it is also better if it is based on each growth stage of the child.

This study had several limitations and strengths. The first limitation was that the participants of the present study were children of a limited age range living in a limited region of Japan; thus, it is necessary to investigate children in other regions and of other ages in order to generalize the present results. Second, this study does not take into account the effects of physiological diurnal variation; that is, not all participants in this study were measured at the same time of day, and therefore diurnal variations may be reflected in the results. Although diurnal variation has not been studied with the same BIA device as that used in the present study, Andersen et al.^60^ evaluated diurnal variation in measurements from a multi-frequency BIA device (Xitron 4200) and found them to be in the range of 1.1–2.8%. In addition, the impedance method was measured only once in the present study, although the BIA device itself has been reported as being reliable in respect to measurement error^38^. Third, the hydration status of the participants, room temperature and humidity, presence or absence of menarche, and the period of the menstrual cycle, were not measured. The BIA method is considered to be influenced by these factors, which may have had some influences on the present results. Fourth, we did not measure the participants’ stretched body height, as proposed by the International Society for the Advancement of Kinanthropometry. Therefore, the heights in the present study may have been affected by diurnal variation. In contrast, the strengths of this study are that it is a population-based study, that we adopted various statistical analysis methods for comparisons between BIA and DXA, and provide precise results of these comparison.

## Conclusion

In this study, we examined the usefulness of a commercially available bioelectrical impedance device by comparing its results with those obtained using a DXA method. Our results revealed strong linear correlations between BIA and DXA, which confirmed the validity of the BIA-derived parameters calculated in this study. However, we also showed that measurement bias exists in all indices and that it varies with sex and degree of BMI. These findings suggest that the BIA device cannot provide the exact same body composition values as DXA, but that it has sufficient measurement performance to be used longitudinally by individuals for daily health management.

### Supplementary Information

Supplementary Information.

## Data Availability

The datasets are available from the corresponding author on reasonable request.
