# Supplementary material for: Similarities and discrepancies between commercially available bioelectrical impedance analysis system and dual-energy X-ray absorptiometry for body composition assessment in 10–14-year-old children
Source: Sci Rep. 2023 Oct 13;13:17420. doi: 10.1038/s41598-023-44217-0 (PMC10576075; doi:10.1038/s41598-023-44217-0)
Supplement: Supplementary file 1 — Supplementary Information. [file 41598_2023_44217_MOESM1_ESM.docx]

**Supplementary Online Content**

Ohara K, et al., Similarity and discrepancies of a commercially available bioelectrical impedance analysis system for body composition assessment in 10-14-year-old children.

Fig S1. Bland-Altman plot of fat mass, fat-free mass, and percentage of body fat obtained with bioelectrical impedance-based methods and DXA of 1st quartile of BMI.

Fig S1 (continued). Bland-Altman plot of lean mass, bone mineral contents, and body mass obtained with bioelectrical impedance-based methods and DXA of 1st quartile of BMI.

Fig S2. Bland-Altman plot of fat mass, fat-free mass, and percentage of body fat obtained with bioelectrical impedance-based methods and DXA of 2nd quartile of BMI.

Fig S2 (continued). Bland-Altman plot of lean mass, bone mineral contents, and body mass obtained with bioelectrical impedance-based methods and DXA of 2nd quartile of BMI.

Fig S3. Bland-Altman plot of fat mass, fat-free mass, and percentage of body fat obtained with bioelectrical impedance-based methods and DXA of 3rd quartile of BMI.

Fig S3 (continued). Bland-Altman plot of lean mass, bone mineral contents, and body mass obtained with bioelectrical impedance-based methods and DXA of 3rd quartile of BMI.

Fig S4. Bland-Altman plot of fat mass, fat-free mass, and percentage of body fat obtained with bioelectrical impedance-based methods and DXA of 4th quartile of BMI.

Fig S4 (continued). Bland-Altman plot of lean mass, bone mineral contents, and body mass obtained with bioelectrical impedance-based methods and DXA of 4th quartile of BMI.

Fig S5. Bland-Altman plot of fat mass, fat-free mass, and percentage of body fat obtained with bioelectrical impedance-based methods and DXA in 5th grade boys and girls.

Fig S5 (continued). Bland-Altman plot of lean mass, bone mineral contents, and body mass obtained with bioelectrical impedance-based methods and DXA in 5th grade boys and girls.

Fig S6. Bland-Altman plot of fat mass, fat-free mass, and percentage of body fat obtained with bioelectrical impedance-based methods and DXA in 6th grade boys and girls.

Fig S6 (continued). Bland-Altman plot of lean mass, bone mineral contents, and body mass obtained with bioelectrical impedance-based methods and DXA in 6th grade boys and girls.

Fig S7. Bland-Altman plot of fat mass, fat-free mass, and percentage of body fat obtained with bioelectrical impedance-based methods and DXA in 7th grade boys and girls.

Fig S7 (continued). Bland-Altman plot of lean mass, bone mineral contents, and body mass obtained with bioelectrical impedance-based methods and DXA in 7th grade boys and girls.

Fig S8. Bland-Altman plot of fat mass, fat-free mass, and percentage of body fat obtained with bioelectrical impedance-based methods and DXA in 8th grade boys and girls.

Fig S8 (continued). Bland-Altman plot of lean mass, bone mineral contents, and body mass obtained with bioelectrical impedance-based methods and DXA in 8th grade boys and girls.

Table S1. Bland-Altman analysis of fat mass, fat-free mass, percentage of body fat, lean body mass, bone mineral contents, and body mass between BIA and DXA

Table S2. Fat mass, fat-free mass, percentage of body fat, lean body mass, bone mineral contents, and body mass by BMI category in boys.

Table S3. Fat mass, fat-free mass, percentage of body fat, lean body mass, bone mineral contents, and body mass by BMI category in girls.

Table S4. Fat mass, fat-free mass, percentage of body fat, lean body mass, bone mineral contents, and body mass by grade in boys.

Table S5. Fat mass, fat-free mass, percentage of body fat, lean body mass, bone mineral contents, and body mass by grade in girls.

Fig S1. Bland-Altman plot of fat mass, fat-free mass, and percentage of body fat obtained with bioelectrical impedance-based methods and DXA in boys and girls of 1st quartile of BMI. (A-1-1) fat mass in boys, (A-1-2) fat-free mass in boys, (A-1-3) percentage of body fat in boys, (B-1-1) fat mass in girls, (B-1-2) fat-free mass in girls, (B-1-3) percentage of body fat in girls.

BIA: bioelectrical impedance analysis, DXA: dual energy X-ray absorptiometry, BMI: body mass index, FM: fat mass, FFM: fat-free mass, PBF: percentage of body fat.

Fig S1 (continued). Bland-Altman plot of lean mass, bone mineral contents, and body mass obtained with bioelectrical impedance-based methods and DXA in boys and girls of 1st quartile of BMI. (A-1-4) lean body mass in boys, (A-1-5) bone mineral contents in boys, (A-1-6) body mass in boys, (B-1-4) lean body mass in girls, (B-1-5) bone mineral contents in girls, (B-1-6) body mass in girls.

BIA: bioelectrical impedance analysis, DXA: dual energy X-ray absorptiometry, BMI: body mass index, LBM: lean body mass, BMC, bone mineral contents, BM: body mass.

Fig S2. Bland-Altman plot of fat mass, fat-free mass, and percentage of body fat obtained with bioelectrical impedance-based methods and DXA in boys and girls of 2nd quartile of BMI. (A-2-1) fat mass in boys, (A-2-2) fat-free mass in boys, (A-2-3) percentage of body fat in boys, (B-2-1) fat mass in girls, (B-2-2) fat-free mass in girls, (B-2-3) percentage of body fat in girls.

BIA: bioelectrical impedance analysis, DXA: dual energy X-ray absorptiometry, BMI: body mass index, FM: fat mass, FFM: fat-free mass, PBF: percentage of body fat.

Fig S2 (continued). Bland-Altman plot of lean mass, bone mineral contents, and body mass obtained with bioelectrical impedance-based methods and DXA in boys and girls of 2nd quartile of BMI. (A-2-4) lean body mass in boys, (A-2-5) bone mineral contents in boys, (A-2-6) body mass in boys, (B-2-4) lean body mass in girls, (B-2-5) bone mineral contents in girls, (B-2-6) body mass in girls.

BIA: bioelectrical impedance analysis, DXA: dual energy X-ray absorptiometry, BMI: body mass index, LBM: lean body mass, BMC, bone mineral contents, BM: body mass.

l contents, BW: body weight.

Fig S3. Bland-Altman plot of body composition data obtained with bioelectrical impedance-based methods and DXA in boys and girls of 3rd quartile of BMI. (A-3-1) fat mass in boys, (A-3-2) fat-free mass in boys, (A-3-3) percentage of body fat in boys, (B-3-1) fat mass in girls, (B-3-2) fat-free mass in girls, (B-3-3) percentage of body fat in girls.

BIA: bioelectrical impedance analysis, DXA: dual energy X-ray absorptiometry, BMI: body mass index, FM: fat mass, FFM: fat-free mass, PBF: percentage of body fat.

Fig S3 (continued). Bland-Altman plot of lean mass, bone mineral contents, and body mass obtained with bioelectrical impedance-based methods and DXA in boys and girls of 3rd quartile of BMI. (A-3-4) lean body mass in boys, (A-3-5) bone mineral contents in boys, (A-3-6) body mass in boys, (B-3-4) lean body mass in girls, (B-3-5) bone mineral contents in girls, (B-3-6) body mass in girls.

BIA: bioelectrical impedance analysis, DXA: dual energy X-ray absorptiometry, BMI: body mass index, LBM: lean body mass, BMC, bone mineral contents, BM: body mass.

Fig S4. Bland-Altman plot of body composition data obtained with bioelectrical impedance-based methods and DXA in boys and girls of 4th quartile of BMI. (A-4-1) fat mass in boys, (A-4-2) fat-free mass in boys, (A-4-3) percentage of body fat in boys, (B-4-1) fat mass in girls, (B-4-2) fat-free mass in girls, (B-4-3) percentage of body fat in girls.

BIA: bioelectrical impedance analysis, DXA: dual energy X-ray absorptiometry, BMI: body mass index, FM: fat mass, FFM: fat-free mass, PBF: percentage of body fat.

Fig S4 (continued). Bland-Altman plot of lean mass, bone mineral contents, and body mass obtained with bioelectrical impedance-based methods and DXA in boys and girls of 4th quartile of BMI. (A-4-4) lean body mass in boys, (A-4-5) bone mineral contents in boys, (A-4-6) body mass in boys, (B-4-4) lean body mass in girls, (B-4-5) bone mineral contents in girls, (B-4-6) body mass in girls.

BIA: bioelectrical impedance analysis, DXA: dual energy X-ray absorptiometry, BMI: body mass index, LBM: lean body mass, BMC, bone mineral contents, BM: body mass.

Fig S5. Bland-Altman plot of body composition data obtained with bioelectrical impedance-based methods and DXA in 5th grade boys and girls. (A-5-1) fat mass in boys, (A-5-2) fat-free mass in boys, (A-5-3) percentage of body fat in boys, (B-5-1) fat mass in girls, (B-5-2) fat-free mass in girls, (B-5-3) percentage of body fat in girls.

BIA: bioelectrical impedance analysis, DXA: dual energy X-ray absorptiometry, BMI: body mass index, FM: fat mass, FFM: fat-free mass, PBF: percentage of body fat.

Fig S5 (continued). Bland-Altman plot of lean mass, bone mineral contents, and body mass obtained with bioelectrical impedance-based methods and DXA in 5th grade boys and girls. (A-5-4) lean body mass in boys, (A-5-5) bone mineral contents in boys, (A-5-6) body mass in boys, (B-5-4) lean body mass in girls, (B-5-5) bone mineral contents in girls, (B-5-6) body mass in girls.

BIA: bioelectrical impedance analysis, DXA: dual energy X-ray absorptiometry, BMI: body mass index, LBM: lean body mass, BMC, bone mineral contents, BM: body mass.

Fig S6. Bland-Altman plot of body composition data obtained with bioelectrical impedance-based methods and DXA in 6th grade boys and girls. (A-6-1) fat mass in boys, (A-6-2) fat-free mass in boys, (A-6-3) percentage of body fat in boys, (B-6-1) fat mass in girls, (B-6-2) fat-free mass in girls, (B-6-3) percentage of body fat in girls.

BIA: bioelectrical impedance analysis, DXA: dual energy X-ray absorptiometry, BMI: body mass index, FM: fat mass, FFM: fat-free mass, PBF: percentage of body fat.

Fig S6 (continued). Bland-Altman plot of lean mass, bone mineral contents, and body mass obtained with bioelectrical impedance-based methods and DXA in 6th grade boys and girls. (A-6-4) lean body mass in boys, (A-6-5) bone mineral contents in boys, (A-6-6) body mass in boys, (B-6-4) lean body mass in girls, (B-6-5) bone mineral contents in girls, (B-6-6) body mass in girls.

BIA: bioelectrical impedance analysis, DXA: dual energy X-ray absorptiometry, BMI: body mass index, LBM: lean body mass, BMC, bone mineral contents, BM: body mass.

Fig S7. Bland-Altman plot of body composition data obtained with bioelectrical impedance-based methods and DXA in 7th grade boys and girls. (A-7-1) fat mass in boys, (A-7-2) fat-free mass in boys, (A-7-3) percentage of body fat in boys, (B-7-1) fat mass in girls, (B-7-2) fat-free mass in girls, (B-7-3) percentage of body fat in girls.

BIA: bioelectrical impedance analysis, DXA: dual energy X-ray absorptiometry, BMI: body mass index, FM: fat mass, FFM: fat-free mass, PBF: percentage of body fat.

Fig S7 (continued). Bland-Altman plot of lean mass, bone mineral contents, and body mass obtained with bioelectrical impedance-based methods and DXA in 7th grade boys and girls. (A-7-4) lean body mass in boys, (A-7-5) bone mineral contents in boys, (A-7-6) body mass in boys, (B-7-4) lean body mass in girls, (B-7-5) bone mineral contents in girls, (B-7-6) body mass in girls.

BIA: bioelectrical impedance analysis, DXA: dual energy X-ray absorptiometry, BMI: body mass index, LBM: lean body mass, BMC, bone mineral contents, BM: body mass.

Fig S8. Bland-Altman plot of body composition data obtained with bioelectrical impedance-based methods and DXA in 8th grade boys and girls. (A-8-1) fat mass in boys, (A-8-2) fat-free mass in boys, (A-8-3) percentage of body fat in boys, (B-8-1) fat mass in girls, (B-8-2) fat-free mass in girls, (B-8-3) percentage of body fat in girls.

BIA: bioelectrical impedance analysis, DXA: dual energy X-ray absorptiometry, BMI: body mass index, FM: fat mass, FFM: fat-free mass, PBF: percentage of body fat.

Fig S8 (continued). Bland-Altman plot of lean mass, bone mineral contents, and body mass obtained with bioelectrical impedance-based methods and DXA in 8th grade boys and girls. (A-8-4) lean body mass in boys, (A-8-5) bone mineral contents in boys, (A-8-6) body mass in boys, (B-8-4) lean body mass in girls, (B-8-5) bone mineral contents in girls, (B-8-6) body mass in girls.

BIA: bioelectrical impedance analysis, DXA: dual energy X-ray absorptiometry, BMI: body mass index, LBM: lean body mass, BMC, bone mineral contents, BM: body mass.

| **Table S1.** Bland-Altman analysis of fat mass, fat-free mass, percentage of body fat, lean body mass, bone mineral contents, and body mass between BIA and DXA. | | | | | |
| --- | --- | --- | --- | --- | --- |
|  | Bland-Altman | | | | |
|  | Bias | Limit of agreement | p value  (one-sample t test) | Function | p value (regression analysis) |
| **Boys (n = 226)** |  |  |  |  |  |
| FM (kg) | -0.28 | -4.93 to 4.36 | 0.076 | y = 0.347x – 2.977 | <0.001 |
| FFM (kg) | 0.07 | -3.86 to 4.01 | 0.584 | y= –0.152x + 5.500 | <0.001 |
| PBF (%) | -1.57 | -10.33 to 7.18 | <0.001 | y= 0.330x – 7.124 | <0.001 |
| LBM (kg) | -0.63 | -4.71 to 3.46 | <0.001 | y= –0.181x + 5.579 | <0.001 |
| BMC (kg) | 0.70 | 0.30 to 1.11 | <0.001 | y= 0.399x + 0.116 | <0.001 |
| BM (kg) | -0.21 | -1.15 to 0.73 | <0.001 | y= 0.046x – 1.375 | <0.001 |
|  |  |  |  |  |  |
| **Girls (n = 217)** |  |  |  |  |  |
| FM (kg) | 0.01 | -2.43 to 2.44 | 0.929 | y = 0.172x – 1.612 | <0.001 |
| FFM (kg) | -0.36 | -2.50 to 1.78 | <0.001 | y= –0.140x + 4.243 | <0.001 |
| PBF (%) | -0.18 | -5.69 to 5.33 | 0.349 | y= 0.199x – 4.448 | <0.001 |
| LBM (kg) | -1.11 | -3.36 to 1.14 | <0.001 | y= –0.175x + 4.383 | <0.001 |
| BMC (kg) | 0.76 | 0.43 to 1.09 | <0.001 | y= 0.399x + 0.116 | <0.001 |
| BM (kg) | -0.35 | -0.98 to 0.28 | <0.001 | y= 0.031x – 1.146 | <0.001 |
|  |  |  |  |  |  |
| **All (n = 443)** |  |  |  |  |  |
| FM (kg) | -0.14 | -3.88 to 3.60 | 0.124 | y = 0.284x – 2.574 | <0.001 |
| FFM (kg) | -0.14 | -3.35 to 3.07 | 0.075 | y= –0.137x + 4.558 | <0.001 |
| PBF (%) | -0.89 | -8.35 to 6.57 | <0.001 | y= 0.286x – 0.6356 | <0.001 |
| LBM (kg) | -0.87 | -4.21 to 2.48 | <0.001 | y= –0.164x + 4.515 | <0.001 |
| BMC (kg) | 0.73 | 0.36 to 1.10 | <0.001 | y= 0.399x + 0.116 | <0.001 |
| BM (kg) | -0.28 | -1.09 to 0.54 | <0.001 | y= 0.041x – 1.323 | <0.001 |
| BIA: bioelectrical impedance analysis, DXA: dual energy X-ray absorptiometry, FM: fat mass, FFM: fat- free mass, PBF: percentage of body fat, LBM: lean body mass, BMC: bone mineral contents, BM: body mass. | | | | | |

| **Table S2.** Fat mass, fat-free mass, percentage of body fat, lean body mass, bone mineral contents, and body mass by BMI category in boys. | | | | | | | |
| --- | --- | --- | --- | --- | --- | --- | --- |
|  | BIA | DXA | CE | SEE | r | ICC (2.1) | CCC (ρ_c_) |
| **Boys, 1st quartile BMI (n = 56) (BMI, 15.2 ± 0.7 kg/m^2^)** | | | | | | | |
| FM (kg) | 2.8 ± 0.7 | 4.8 ± 1.1 | -1.90 ± 0.77 | 1.492 | 0.639^*^ | 0.171 | 0.169 |
| FFM (kg) | 28.9 ± 3.7 | 27.5 ± 3.6 | 1.46 ± 0.77 | 1.571 | 0.978^*^ | 0.905 | 0.903 |
| PBF (%) | 9.0 ± 2.1 | 14.8 ± 2.9 | -5.81 ± 2.31 | 3.003 | 0.616^*^ | 0.159 | 0.202 |
| LBM (kg) | 27.5 ± 3.4 | 26.6 ± 3.5 | 0.90 ± 0.75 | 1.523 | 0.977^*^ | 0.945 | 0.944 |
| BMC (kg) | 1.42 ± 0.23 | 0.86 ± 0.15 | 0.56 ± 0.13 | 0.130 | 0.864^*^ | 0.148 | 0.145 |
| BM (kg) | 31.8 ± 3.8 | 32.2 ± 3.8 | -0.45 ± 0.18 | 0.336 | 0.999^*^ | 0.992 | 0.992 |
|  |  |  |  |  |  |  |  |
| **Boys, 2nd quartile BMI (n = 57) (BMI, 17.0 ± 0.5 kg/m^2^)** | | | | | | | |
| FM (kg) | 4.8 ± 1.1 | 6.0 ± 1.4 | -1.20 ± .78 | 1.433 | 0.833^*^ | 0.552 | 0.548 |
| FFM (kg) | 34.1 ± 5.1 | 33.3 ± 5.3 | 0.77 ± 0.76 | 1.203 | 0.990^*^ | 0.978 | 0.978 |
| PBF (%) | 12.4 ± 3.0 | 15.4 ± 3.9 | -2.98 ± 2.02 | 2.877 | 0.857^*^ | 0.602 | 0.658 |
| LBM (kg) | 32.4 ± 4.8 | 32.3 ± 5.1 | 0.10 ± 0.76 | 1.189 | 0.989^*^ | 0.988 | 0.988 |
| BMC (kg) | 1.73 ± 0.31 | 1.05 ± 0.21 | 0.68 ± 0.17 | 0.140 | 0.869^*^ | 0.190 | 0.187 |
| BM (kg) | 38.9 ± 5.2 | 39.3 ± 5.2 | -0.42 ± 0.19 | 0.245 | 0.999^*^ | 0.996 | 0.996 |
|  |  |  |  |  |  |  |  |
| **Boys, 3rd quartile BMI (n = 57) (BMI, 18.7 ± 0.6 kg/m^2^)** | | | | | | | |
| FM (kg) | 7.1 ± 1.4 | 7.5 ± 1.8 | -0.39 ± 1.20 | 1.059 | 0.754^*^ | 0.710 | 0.707 |
| FFM (kg) | 37.3 ± 6.4 | 37.2 ± 7.1 | -0.15 ± 1.19 | 1.442 | 0.990^*^ | 0.985 | 0.984 |
| PBF (%) | 16.3 ± 3.7 | 17.3 ± 5.4 | -1.01 ± 2.64 | 2.649 | 0.894^*^ | 0.820 | 0.799 |
| LBM (kg) | 35.4 ± 6.0 | 36.0 ± 6.0 | -0.59 ± 1.25 | 1.420 | 0.990^*^ | 0.978 | 0.977 |
| BMC (kg) | 1.93 ± 0.39 | 1.18 ± 0.27 | 0.74 ± 0.19 | 0.088 | 0.888^*^ | 0.238 | 0.235 |
| BM (kg) | 44.4 ± 6.6 | 4.7 ± 6.6 | -0.24 ± 0.24 | 0.298 | 0.999^*^ | 0.999 | 0.999 |
|  |  |  |  |  |  |  |  |
| **Boys, 4th quartile BMI (n = 56) (BMI, 22.8 ± 3.1 kg/m^2^)** | | | | | | | |
| FM (kg) | 15.8 ± 7.3 | 13.4 ± 5.2 | 2.39 ± 3.07 | 1.313 | 0.932^*^ | 0.826 | 0.823 |
| FFM (kg) | 42.5 ± 7.3 | 44.6 ± 8.1 | -2.10 ± 2.57 | 2.149 | 0.951^*^ | 0.912 | 0.910 |
| PBF (%) | 23.1 ± 7.5 | 23.1 ± 7.5 | -3.53 ± 4.22 | 2.745 | 0.879^*^ | 0.795 | 0.809 |
| LBM (kg) | 40.3 ± 6.8 | 43.2 ± 7.9 | -2.93 ± 2.58 | 2.128 | 0.948^*^ | 0.871 | 0.869 |
| BMC (kg) | 2.24 ± 0.44 | 1.41 ± 0.31 | 0.83 ± 0.22 | 0.086 | 0.883^*^ | 0.241 | 0.238 |
| BM (kg) | 58.4 ± 10.2 | 58.1 ± 9.7 | 0.29 ± 0.68 | 0.257 | 0.999^*^ | 0.997 | 0.997 |
| Data are mean ± standard deviation. *p < 0.05 (Pearson’s correlation coefficient)  BIA: bioelectrical impedance analysis, DXA: dual energy X-ray absorptiometry, CE: constant error, SEE: standard error of estimate, r: Pearson’s correlation coefficient, ICC: intraclass correlation coefficients, CCC: concordance correlation coefficients, FM: fat mass, FFM: fat-free mass, PBF: percentage of body fat, BMC: bone mineral content, BM: body mass. | | | | | | | |

| **Table S3.** Fat mass, fat-free mass, percentage of body fat, lean body mass, bone mineral contents, and body mass by BMI category in girls. | | | | | | | |
| --- | --- | --- | --- | --- | --- | --- | --- |
|  | BIA | DXA | CE | SEE | r | ICC (2.1) | CCC (ρ_c_) |
| **Girls, 1st quartile BMI (n = 54) (BMI, 15.5 ± 1.0 kg/m^2^)** | | | | | | | |
| FM (kg) | 5.1 ± 1.3 | 6.0 ± 1.2 | -0.86 ± 0.66 | 1.207 | 0.863^*^ | 0.689 | 0.685 |
| FFM (kg) | 28.3 ± 3.5 | 28.0 ± 3.9 | 0.32 ± 0.67 | 0.966 | 0.988^*^ | 0.980 | 0.980 |
| PBF (%) | 15.1 ± 2.6 | 17.5 ± 2.5 | -2.49 ± 2.16 | 2.180 | 0.653^*^ | 0.448 | 0.508 |
| LBM (kg) | 26.8 ± 3.3 | 27.1 ± 3.7 | -0.32 ± 0.71 | 0.954 | 0.987^*^ | 0.976 | 0.975 |
| BMC (kg) | 1.57 ± 0.27 | 0.92 ± 0.18 | 0.65 ± 0.14 | 0.133 | 0.869^*^ | 0.161 | 0.158 |
| BM (kg) | 33.4 ± 4.5 | 34.0 ± 4.5 | -0.54 ± 0.26 | 0.230 | 0.998^*^ | 0.991 | 0.991 |
|  |  |  |  |  |  |  |  |
| **Girls, 2nd quartile BMI (n = 54) (BMI, 17.3 ± 0.4 kg/m^2^)** | | | | | | | |
| FM (kg) | 7.3 ± 13 | 7.8 ± 1.4 | -0.43 ± 0.93 | 1.283 | 0.770^*^ | 0.732 | 0.729 |
| FFM (kg) | 31.8 ± 3.4 | 31.9 ± 3.8 | -0.01 ± 0.84 | 1.069 | 0.979^*^ | 0.974 | 0.973 |
| PBF (%) | 18.7 ± 2.4 | 19.7 ± 3.5 | -0.99 ± 2.36 | 2.299 | 0.737^*^ | 0.657 | 0.636 |
| LBM (kg) | 30.0 ± 3.1 | 30.8 ± 3.6 | -0.76 ± 0.87 | 1.052 | 0.978^*^ | 0.944 | 0.943 |
| BMC (kg) | 1.82 ± 0.25 | 1.07 ± 0.19 | 0.76 ± 0.14 | 0.105 | 0.838^*^ | 0.120 | 0.118 |
| BM (kg) | 39.2 ± 4.1 | 39.6 ± 4.1 | -0.44 ± 0.26 | 0.239 | 0.998^*^ | 0.992 | 0.992 |
|  |  |  |  |  |  |  |  |
| **Girls, 3rd quartile BMI (n = 55) (BMI, 19.0 ± 0.6 kg/m^2^)** | | | | | | | |
| FM (kg) | 10.1 ± 1.4 | 9.8 ± 1.6 | 0.36 ± 0.95 | 0.910 | 0.809^*^ | 0.779 | 0.776 |
| FFM (kg) | 34.1 ± 2.7 | 34.9 ± 3.1 | -0.72 ± 0.85 | 0.664 | 0.966^*^ | 0.931 | 0.929 |
| PBF (%) | 22.9 ± 2.2 | 21.9 ± 3.2 | 0.96 ± 2.05 | 2.121 | 0.764^*^ | 0.678 | 0.665 |
| LBM (kg) | 32.1 ± 2.5 | 33.6 ± 3.0 | -1.47 ± 0.87 | 0.655 | 0.963^*^ | 0.833 | 0.830 |
| BMC (kg) | 2.00 ± 0.21 | 1.24 ± 0.19 | 0.76 ± 0.15 | 0.106 | 0.734^*^ | 0.091 | 0.089 |
| BM (kg) | 44.3 ± 3.6 | 44.6 ± 3.6 | -0.36 ± 0.27 | 0.329 | 0.997^*^ | 0.992 | 0.992 |
|  |  |  |  |  |  |  |  |
| **Girls, 4th quartile BMI (n = 54) (BMI, 21.7 ± 1.6 kg/m^2^)** | | | | | | | |
| FM (kg) | 15.0 ± 3.3 | 14.1 ± 2.9 | 0.96 ± 1.44 | 0.954 | 0.902^*^ | 0.855 | 0.852 |
| FFM (kg) | 36.8 ± 3.5 | 37.8 ± 4.1 | -1.03 ± 1.33 | 0.702 | 0.950^*^ | 0.907 | 0.906 |
| PBF (%) | 28.8 ± 4.0 | 27.0 ± 4.1 | 1.78 ± 2.52 | 2.000 | 0.812^*^ | 0.744 | 0.775 |
| LBM (kg) | 34.6 ± 3.2 | 36.5 ± 3.9 | -1.90 ± 1.35 | 0.667 | 0.947^*^ | 0.817 | 0.815 |
| BMC (kg) | 2.20 ± 0.28 | 1.33 ± 0.23 | 0.88 ± 0.17 | 0.106 | 0.802^*^ | 0.112 | 0.110 |
| BM (kg) | 51.8 ± 5.6 | 51.9 ± 5.5 | -0.07 ± 0.30 | 0.241 | 0.999^*^ | 0.999 | 0.998 |
| Data are mean ± standard deviation. *p < 0.05 (Pearson’s correlation coefficient)  BIA: bioelectrical impedance analysis, DXA: dual energy X-ray absorptiometry, CE: constant error, SEE: standard error of estimate, r: Pearson’s correlation coefficient, ICC: intraclass correlation coefficients, CCC: concordance correlation coefficients, FM: fat mass, FFM: fat-free mass, PBF: percentage of body fat, BMC: bone mineral content, BM: body mass. | | | | | | | |

| **Table S4.** Fat mass, fat-free mass, percentage of body fat, lean body mass, bone mineral contents, and body mass by grade in boys. | | | | | | | |
| --- | --- | --- | --- | --- | --- | --- | --- |
|  | BIA | DEXA | CE | SEE | r | ICC (2.1) | CCC |
| **Boys, 5th grade (n = 55) (Age, 10.7 ± 0.5 yrs)** | | | |  |  |  |  |
| FM (kg) | 6.8 ± 5.8 | 7.7 ± 4.2 | -0.88 ± 2.00 | 1.492 | 0.969^*^ | 0.909 | 0.908 |
| FFM (kg) | 29.1 ± 3.1 | 28.5 ± 4.3 | 0.57 ± 1.69 | 1.571 | 0.942^*^ | 0.889 | 0.887 |
| PBF(%) | 17.0 ± 9.2 | 20.1 ± 6.3 | -3.12 ± 4.35 | 3.003 | 0.908^*^ | 0.787 | 0.816 |
| LBM (kg/m^2^) | 27.7 ± 2.9 | 27.6 ± 4.1 | 0.03 ± 1.71 | 1.523 | 0.941^*^ | 0.889 | 0.887 |
| BMC (kg) | 1.4 ± 0.2 | 0.9 ± 0.1 | 0.55 ± 0.11 | 0.130 | 0.813^*^ | 0.128 | 0.126 |
| BM (kg) | 35.8 ± 8.2 | 36.2 ± 7.9 | -0.32 ± 0.41 | 0.336 | 0.999^*^ | 0.998 | 0.998 |
|  |  |  |  |  |  |  |  |
| **Boys, 6th grade (n = 53) (Age, 11.5 ± 0.5 yrs)** | | | |  |  |  |  |
| FM (kg) | 6.9 ± 6.6 | 7.7 ± 4.6 | -0.82 ± 2.51 | 1.433 | 0.965^*^ | 0.895 | 0.893 |
| FFM (kg) | 32.0 ± 4.9 | 31.5 ± 5.8 | 0.50 ± 2.21 | 1.203 | 0.931^*^ | 0.913 | 0.912 |
| PBF(%) | 15.7 ± 9.2 | 18.6 ± 6.2 | -2.88 ± 4.52 | 2.877 | 0.898^*^ | 0.783 | 0.815 |
| LBM (kg/m^2^) | 30.4 ± 4.6 | 30.5 ± 5.7 | -0.13 ± 2.22 | 1.189 | 0.928^*^ | 0.909 | 0.907 |
| BMC (kg) | 1.6 ± 0.3 | 1.0 ± 0.2 | 0.63 ± 0.15 | 0.140 | 0.901^*^ | 0.202 | 0.199 |
| BM (kg) | 38.8 ± 9.7 | 39.1 ± 9.4 | -0.32 ± 0.38 | 0.245 | 1.000^*^ | 0.999 | 0.999 |
|  |  |  |  |  |  |  |  |
| **Boys, 7th grade (n = 63) (Age, 12.6 ± 0.5 yrs)** | | | |  |  |  |  |
| FM (kg) | 9.2 ± 7.7 | 8.8 ± 5.5 | 0.42 ± 2.73 | 1.059 | 0.967^*^ | 0.916 | 0.915 |
| FFM (kg) | 38.8 ± 6.9 | 39.3 ± 8.6 | -0.49 ± 2.20 | 1.442 | 0.983^*^ | 0.958 | 0.958 |
| PBF(%) | 17.2 ± 9.1 | 17.3 ± 6.5 | -0.12 ± 4.43 | 2.649 | 0.890^*^ | 0.845 | 0.865 |
| LBM (kg/m^2^) | 36.8 ± 6.5 | 38.1 ± 8.3 | -1.28 ± 2.30 | 1.420 | 0.983^*^ | 0.939 | 0.938 |
| BMC (kg) | 2.0 ± 0.4 | 1.2 ± 0.3 | 0.79 ± 0.22 | 0.088 | 0.885^*^ | 0.234 | 0.231 |
| BM (kg) | 48.1 ± 13.4 | 48.1 ± 12.8 | -0.07 ± 0.65 | 0.298 | 1.000^*^ | 0.999 | 0.999 |
|  |  |  |  |  |  |  |  |
| **Boys, 8th grade (n = 55) (Age, 13.6 ± 0.5 yrs)** | | | |  |  |  |  |
| FM (kg) | 7.4 ± 3.5 | 7.3 ± 2.5 | 0.04 ± 1.85 | 1.313 | 0.863^*^ | 0.822 | 0.819 |
| FFM (kg) | 42.4 ± 6.0 | 42.6 ± 7.3 | -0.19 ± 1.67 | 2.149 | 0.986^*^ | 0.969 | 9.968 |
| PBF(%) | 14.1 ± 4.8 | 14.5 ± 3.7 | -0.43 ± 3.75 | 2.745 | 0.644^*^ | 0.624 | 0.649 |
| LBM (kg/m^2^) | 40.2 ± 5.6 | 41.2 ± 7.0 | -1.02 ± 1.74 | 2.128 | 0.986^*^ | 0.951 | 0.950 |
| BMC (kg) | 12.2 ± 0.4 | 1.4 ± 0.3 | 0.84 ± 0.17 | 0.086 | 0.879^*^ | 0.199 | 0.196 |
| BM (kg) | 49.8 ± 8.9 | 49.9 ± 8.7 | -0.15 ± 0.35 | 0.257 | 1.000^*^ | 0.999 | 0.999 |
| Data are mean ± standard deviation. *p < 0.05 (Pearson’s correlation coefficient)  BIA: bioelectrical impedance analysis, DXA: dual energy X-ray absorptiometry, CE: constant error, SEE: standard error of estimate, r: Pearson’s correlation coefficient, ICC: intraclass correlation coefficients, CCC: concordance correlation coefficients, FM: fat mass, FFM: fat-free mass, PBF: percentage of body fat, BMC: bone mineral content, BM: body mass. | | | | | | | |

| **Table S5.** Fat mass, fat-free mass, percentage of body fat, lean body mass, bone mineral contents, and body mass by grade in girls. | | | | | | | |
| --- | --- | --- | --- | --- | --- | --- | --- |
|  | BIA | DEXA | CE | SEE | r | ICC (2.1) | CCC |
| **Girls, 5th grade (n = 48) (Age, 10.6 ± 0.5 yrs)** | | | |  |  |  |  |
| FM (kg) | 7.4 ± 3.7 | 8.1 ± 3.3 | -0.72 ± 0.91 | 1.207 | 0.972^*^ | 0.946 | 0.945 |
| FFM (kg) | 28.7 ± 4.1 | 28.4 ± 4.5 | 0.26 ± 0.73 | 0.966 | 0.989^*^ | 0.984 | 0.984 |
| PBF(%) | 19.5 ± 5.9 | 21.1 ± 5.0 | -0.43 ± 2.54 | 2.180 | 0.906^*^ | 0.835 | 0.844 |
| LBM (kg/m^2^) | 27.1 ± 3.8 | 27.5 ± 4.3 | -0.43 ± 0.80 | 0.954 | 0.989^*^ | 0.976 | 0.975 |
| BMC (kg) | 1.6 ± 0.3 | 0.9 ± 0.2 | 0.69 ± 0.18 | 0.133 | 0.824^*^ | 0.153 | 0.150 |
| BM (kg) | 36.0 ± 7.2 | 36.5 ± 7.1 | -0.46 ± 0.37 | 0.230 | 0.999^*^ | 0.997 | 0.997 |
|  |  |  |  |  |  |  |  |
| **Girls, 6th grade (n = 50) (Age, 11.5 ± 0.5 yrs)** | | | |  |  |  |  |
| FM (kg) | 9.2 ± 4.5 | 9.0 ± 3.7 | 0.17 ± 1.13 | 1.283 | 0.980^*^ | 0.962 | 0.961 |
| FFM (kg) | 32.1 ± 4.3 | 32.6 ± 5.1 | -0.52 ± 1.01 | 1.069 | 0.991^*^ | 0.972 | 0.971 |
| PBF(%) | 21.1 ± 6.5 | 21.0 ± 4.9 | 0.14 ± 2.86 | 2.299 | 0.913^*^ | 0.878 | 0.897 |
| LBM (kg/m^2^) | 30.3 ± 4.0 | 31.5 ± 4.9 | -1.27 ± 1.09 | 1.052 | 0.991^*^ | 0.933 | 0.932 |
| BMC (kg) | 1.8 ± 0.3 | 1.1 ± 0.2 | 0.75 ± 0.17 | 0.105 | 0.881^*^ | 0.179 | 0.176 |
| BM (kg) | 41.3 ± 8.3 | 41.7 ± 8.2 | -0.36 ± 0.27 | 0.239 | 1.000^*^ | 0.999 | 0.999 |
|  |  |  |  |  |  |  |  |
| **Girls, 7th grade (n = 73) (Age, 12.6 ± 0.5 yrs)** | | | |  |  |  |  |
| FM (kg) | 9.9 ± 3.7 | 9.9 ± 3.3 | -0.08 ± 1.10 | 0.910 | 0.955^*^ | 0.950 | 0.950 |
| FFM (kg) | 34.3 ± 3.5 | 34.5 ± 3.9 | -0.29 ± 1.00 | 0.664 | 0.969^*^ | 0.961 | 0.961 |
| PBF(%) | 21.8 ± 5.2 | 21.9 ± 4.9 | -0.16 ± 2.33 | 2.121 | 0.896^*^ | 0.895 | 0.894 |
| LBM (kg/m^2^) | 32.3 ± 3.2 | 33.3 ± 3.7 | -1.06 ± 1.02 | 0.655 | 0.967^*^ | 0.916 | 0.914 |
| BMC (kg) | 2.0 ± 0.3 | 1.2 ± 0.2 | 0.78 ± 0.16 | 0.106 | 0.815^*^ | 0.138 | 0.137 |
| BM (kg) | 44.1 ± 6.5 | 44.5 ± 6.3 | -0.37 ± 0.29 | 0.329 | 0.999^*^ | 0.997 | 0.997 |
|  |  |  |  |  |  |  |  |
| **Girls, 8th grade (n = 46) (Age, 13.6 ± 0.5 yrs)** | | | |  |  |  |  |
| FM (kg) | 11.0 ± 4.5 | 10.3 ± 3.7 | 0.73 ± 1.43 | 0.954 | 0.956^*^ | 0.926 | 0.924 |
| FFM (kg) | 35.4 ± 3.3 | 36.4 ± 4.2 | -0.94 ± 1.28 | 0.702 | 0.968^*^ | 0.916 | 0.914 |
| PBF(%) | 23.0 ± 5.7 | 21.6 ± 4.9 | 1.41 ± 2.67 | 2.000 | 0.885^*^ | 0.847 | 0.857 |
| LBM (kg/m^2^) | 33.4 ± 3.1 | 335.1 ± 4.1 | -1.74 ± 1.33 | 0.667 | 0.966^*^ | 0.835 | 0.831 |
| BMC (kg) | 2.1 ± 0.3 | 1.3 ± 0.2 | 0.81 ± 0.14 | 0.106 | 0.826^*^ | 0.102 | 0.100 |
| BM (kg) | 46.5 ± 7.2 | 46.7 ± 7.0 | -0.21 ± 0.32 | 0.241 | 0.999^*^ | 0.999 | 0.999 |
| Data are mean ± standard deviation. *p < 0.05 (Pearson’s correlation coefficient)  BIA: bioelectrical impedance analysis, DXA: dual energy X-ray absorptiometry, CE: constant error, SEE: standard error of estimate, r: Pearson’s correlation coefficient, ICC: intraclass correlation coefficients, CCC: concordance correlation coefficients, FM: fat mass, FFM: fat-free mass, PBF: percentage of body fat, BMC: bone mineral content, BM: body mass. | | | | | | | |
